# The Digital Future of Heart Failure Care

**DOI:** 10.1007/s11897-022-00547-0

**Published:** 2022-04-19

**Authors:** M. R. Cowie, K. C. C. McBeath, C. E. Angermann

**Affiliations:** 1 Royal Brompton Hospital (Guy’s & St Thomas’ NHS Foundation Trust), Sydney Street, London, SW3 6NP UK; 2 Faculty of Medicine & Lifesciences, School of Cardiovascular Medicine, King’s College London, London, UK; 3 Comprehensive Heart Failure Centre, University and University Hospital Würzburg, Würzburg, Germany

**Keywords:** Digital Health, Heart failure, Person-centred care, Artificial intelligence, Co-design

## Introduction

**Fig. 1:**
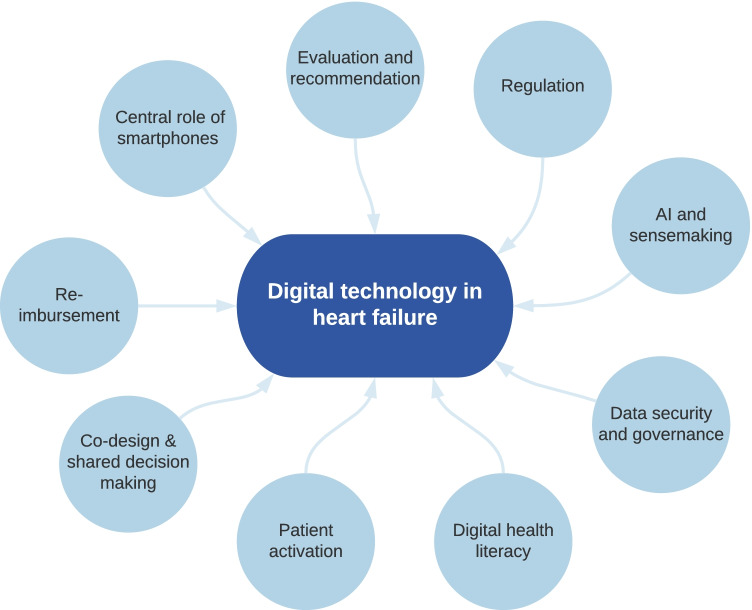
Key factors in the development and adoption of digital technology in heart failure care. AI, artificial intelligence

In our article published in this edition of the journal [1], we review the body of evidence on tele- and remote monitoring in patients with heart failure (HF), including the use of cardiac implantable electronic devices, implantable haemodynamic monitors and wearable digital technology. We discuss the lessons learnt and what a contemporary HF model of care might look like. These technologies and lessons cannot, and should not, be viewed in isolation. They are part of a broader digital health care evolution in a complex and highly regulated healthcare environment. In this commentary, we discuss some of those broader contextual issues, including the evaluation and regulation of technology, data security and the use of artificial intelligence. Vital to success is the appreciation that patients need to be seen in a holistic context, with an understanding of their digital and health needs and capabilities, including their knowledge, skills, attitudes and access. These factors are summarised in Fig. 1.

## The Current Digital Landscape for the Patient and the Healthcare Team

There are many digital technologies and approaches that can be used in the HF pathway [1]. Many of these have been designed and tested “on” patients, rather than “with” patients, and the concept of co-design and co-implementation is only now becoming a clearer success factor for any technology [2, 3]. Co-design is more than merely using feedback from patients to support design, but having them at the centre of the design process [4].

Additionally, instead of a “one size fits all” approach—with a technology being used in a particular setting for all patients—there is a better understanding of the need to assess the patients within their social setting and the healthcare system around them. What are the shared goals of the patients, their family and the healthcare professionals (HCPs)? What are the capabilities and digital and health literacy of each individual patient? How “activated” are patients in engaging with their health and decision-making? Patient activation is defined as an individual’s knowledge, skill, motivation and confidence in managing their health and healthcare; the more activated a patient is, the better the healthcare outcome is likely to be [5]. What would be the most appropriate digital tool to support the shared goals—and what support will be required to optimise its use?

Digital technology should not replace human-to-human contact, but should enrich it, enabling more patients to access appropriate care and support at the right time.

Digital access is not equitable: even in relatively wealthy countries, such as the UK, there are still 5.3 million adults (10% of the adult population) classed as “internet non-users”, i.e. have never used the internet or have not used it in the preceding few months [6]. Age is not necessarily a barrier to digital approaches to care, but with the average age of a patient admitted with HF to European hospitals being around 80 years [7], there is a need to consider, evaluate and strengthen digital health literacy capacities across all age groups.

Using the internet is an important step in increasing digital literacy and providing access to a range of modern digital tools, but even amongst students in German universities, there is a significant proportion who struggles to evaluate the quality of health-related information on the internet [8].

Digital advocacy is increasing within the general population. In the UK, for example, 65% of people believe that it is vital that individuals look at digital methods (including the use of mobile health applications “apps”) to manage their health to support the national healthcare system [9]. Half of those surveyed thought that these apps should be “prescribed”—digital tools should be considered as carefully as a medicine—but as few as 8% of these are [9]. This is likely to affect the quality of the apps selected, and persistence with their use.

It is difficult for HCPs to navigate the digital tool landscape—90,000 new health apps were released in 2020 alone [10]. If the healthcare system is to move to an evidence-based selection of health apps and other smartphone-based technologies, we will need better co-design, more robust assessment and faster regulatory and reimbursement decision-making to support clinicians and the patients they advise.

Regulators, including the US Food and Drug Administration (FDA), have recognised the unmet need for better evaluation and regulation, setting up the *Digital Health Centre of Excellence* in late 2020, with the stated aim of empowering digital health stakeholders to advance health care. Other organisations have also considered the need for more agile and proportionate evaluation and approval of digital technologies, which—unlike a medicine—evolve rapidly. An example of this is the National Institute for Health and Care Excellence (NICE) in England, which has established a collaborative evidence standards framework to help technology companies demonstrate the value of their tools to the UK health- and social care system.

Within the EU, device regulation has been tightened recently, and many digital tools may be reclassified as low risk “health and lifestyle” tools rather than medical devices, but the remainder will be held to account to a greater degree than previously regarding safety and efficacy [11]. The FDA’s digital health innovation plan includes several practical policies to focus on these higher-risk digital tools. These changes should ensure a proportionate use of time and resources and will likely lead to development of pre-certification programmes for trusted developers, particularly with real-world data to show safety and effectiveness can be delivered [12].

Few governmental organisations evaluate a large number of apps from the clinical impact perspective; the FDA only approved 11 digital health products in the first quarter of 2021 (four of which come with an associated patient-facing app [13] and the UK’s NHS App library has recently been decommissioned after a poor turnover of apps. Following the introduction of the Digital Care Act in Germany (permitting reimbursement for the prescription of a limited range of apps), 24 apps have been listed in the directory, leading to over 50,000 prescriptions in routine practice from October 2020 to November 2021 [14].

Commercial organisations, such as ORCHA, are active in this space, and work with British, American and Canadian regulatory bodies. ORCHA has successfully reviewed over 6000 apps [9].

Professional medical associations, including the European Society of Cardiology, the American Heart Association and the American College of Cardiology, are cautious about this rapidly evolving field. Clinical guidelines appear guarded about some of the developments and apply a randomised controlled trial mindset to evaluation, destining many digital technologies and approaches to either be unmentioned or badged with (at best) a Level 2b recommendation of “may be considered”. The risk of this approach is that innovation is stifled, not implemented to scale, and clinical practice is seen to be increasingly divorced from the realities of modern life.

## Managing a Tsunami of Data: Security and Sensemaking

We are amid a rapid surge in data volume and velocity. Thirty percent of the world’s data is produced by the healthcare industry, and it is predicted that the annual growth in healthcare data will reach 36% within 5 years, faster than any other industry [15]. This presents challenges in terms of data security and “sensemaking”.

Despite the tsunami of data, the healthcare industry is amongst the lowest rank for information technology (IT) investment [16]. In the US last year, 40 million people were affected by health data breaches [17], exceeding that related to credit card fraud. Concerns over privacy and data security (and appropriate use of data) are amongst the biggest barriers to patients using digital tools [18]. The EU has set out a “Data Governance Act” to create a new governance structure for data access, sharing and monetisation, the first initiative of their broader data strategy [19].

The volume, velocity and variety of data now available are well beyond human cognitive capacity to interpret. Machine learning techniques used in artificial intelligence (AI) can, however, use this tsunami of data to provide, arguably, more efficient, more personalised and more multidimensional decision-making. Without it, clinicians will be overwhelmed in their attempts to make sense of the increasingly varied and complex data streams from health apps, implantable devices, wearable sensors, genomics and complex imaging. AI can help make sense of these data, supporting better diagnosis, risk stratification and prognostication [20], thus allowing clinicians to make better decisions, and freeing them up to spend their time on activities that require a human touch. Already, AI can provide rapid and accurate analysis of ECGs and cardiac images [21, 22], and is increasingly used in risk stratification and treatment decision-making.

There are concerns about the potential harm of using AI. These include legal liability if a machine-learning approach is used without appropriate oversight or understanding, or of inadvertent introduction of bias into decision-making, particularly where validation of the algorithms has not been done in the relevant population or merely copies human biases. Algorithms that “learn” after initial development are of particular concern. The difficulty for an HCP in explaining how a decision has been arrived at—the so called “black box” of AI—also limits adoption. The EU AI commission [23] seeks to ensure that best practices are followed, with transparency on how clinical prediction models work, and with standardisation of reporting. Such moves are vital for understanding and trust in the results [24]. Professional consensus recommendations are also likely to be helpful, with publication and peer-review of machine learning approaches to clinical decision-making essential [25]. From the ethical point of view, Arnold argues that physicians should neither uncritically accept nor unreasonably resist developments in AI, but should actively engage and contribute to the discussion, since AI will undoubtedly affect their roles and the nature of their work [26].

## Reimbursement, Funding and the Digital Divide

The term “prescription” implies a considered offering of a therapy authorised by a regulatory body as safe and efficacious, and in many countries also implies at least some subsidisation of cost to the patient.

Digital technologies are familiar to most citizens, but within the tightly regulated healthcare environment it can take many years from a new technology being available to it being reimbursed widely.

The COVID-19 pandemic provided an exception to this: healthcare systems and those who funded them rapidly became more permissive of remote monitoring and remote consultation. Consequently, the use and acceptability of remote technologies increased rapidly with, for example, remote consultation almost entirely replacing face-to-face clinic review for patients in HF services during “lockdowns”. In the USA, the Centres for Medicare & Medicaid services permitted reimbursement of remote consultations at a similar rate to face-to-face interactions during lockdowns. In Europe, pathways are now clearer: in Germany, the DiGA programme permits (albeit restricted) reimbursement for remote monitoring of HF [27], as does the French Health Authority [28]. In the UK, there was an innovation tariff to reimburse general practitioner use of ECG monitoring using the Kardia™ device [29], and the prescription of a limited number of apps, including for chronic lung disease [30] and for cognitive behavioural therapy.

There is concern about a “digital divide” opening between those with disposable income to purchase technologies, including apps (and the internet connectivity that underpins their value), and those who cannot or chose not to do so. Supporting those unfamiliar with digital technologies to use these better, and with improved co-design and prescription of technologies, this problem should become less with time.

## Conclusion

Our article, “Digital technologies to support better outcome and experience of care in heart failure patients”, discusses the growth of a substantial body of evidence around remote monitoring in HF care [1]. We highlight the exponential rise of “smarter” digital tools that use multiparametric data and provide a new opportunity to improve clinical outcomes for patients and improve the convenience and accessibility of services.

The digital health landscape is complex and is evolving rapidly. Technical feasibility is necessary, but not sufficient, for adoption at pace and at scale. A broader understanding of the key issues in digital health and earlier engagement of all the stakeholders is essential. As digital health becomes part of the new “normal”, we need to ensure we identify the right tool for the right purpose and optimise its use by consideration of the broader context of the patient, their social setting and digital literacy and the healthcare environment. The co-design of future HF care is an exciting challenge—but ultimately the key question is how we best achieve the shared aims of care. Digital technology will undoubtedly be central to this process but human factors will remain key.
